# Long-term outcomes of S-1 monotherapy in stage IIIA gastric cancer with small tumors and low nodal involvement

**DOI:** 10.1007/s00423-025-03781-x

**Published:** 2025-07-10

**Authors:** Tsunehiko Maruyama, Yoshimasa Akashi, Reiji Nozaki, Yusuke Ozawa, Makoto Uchino, Tatsuya Oda

**Affiliations:** 1 Department of Surgery, Mito Saiseikai General Hospital, 3-3-10 Futabadai, Mito-city, 311-4198 Ibaraki Japan; 2 Department of Gastroenterological Surgery, University of Tsukuba, Tsukuba, Japan

**Keywords:** Stage IIIA gastric cancer, Tumor size, Lymph node metastasis rates, Recurrence-free survival, Adjuvant chemotherapy

## Abstract

**Background:**

In Japan, adjuvant chemotherapy with docetaxel/S-1 is recommended for stage III gastric cancer. However, this regimen may not be suitable for all patients due to toxicity and tolerability issues, particularly in older individuals or those with comorbidities. This study aimed to explore prognostic factors in stage IIIA gastric cancer and assess long-term outcomes in patients treated with S-1 monotherapy after curative gastrectomy.

**Methods:**

We retrospectively analyzed 73 patients with stage IIIA gastric cancer (UICC 7th edition) who underwent curative gastrectomy and received postoperative adjuvant S-1 monotherapy between 2005 and 2018.Various prognostic factors, including preoperative (age, sex, BMI, tumor markers), perioperative (surgical approach, operative duration, blood loss, complications), and tumor-related variables (tumor size, lymph node status, histological features), were analyzed. Recurrence-free survival (RFS) was the primary endpoint, and cut-off values were determined using ROC analysis. Cox proportional hazards models were used for univariate and multivariate analyses.

**Results:**

The 3- and 5-year RFS rates were 71.7% and 64.3%, respectively. Multivariate analysis identified smaller tumor size (≤ 55.0 mm, *P* = 0.006) and lower lymph node positivity rate (≤ 0.079, *P* = 0.008) as independent favorable prognostic factors.

**Conclusion:**

S-1 monotherapy may be associated with favorable long-term outcomes in selected patients with stage IIIA gastric cancer who have small tumors and low lymph node positivity rates. While these results are encouraging, they should not be interpreted as a rationale for broadly recommending S-1 monotherapy. Further studies are needed to define its role in individualized treatment strategies.

## Introduction

Numerous meta-analyses have indicated the survival advantage of adjuvant chemotherapy in gastric cancer patients [1, 2] but its efficacy has been definitively validated in only a limited number of large-scale clinical trials. In the United States, the standard postoperative regimen involves radiotherapy combined with 5-FU and leucovorin [3]whereas the United Kingdom employs a perioperative triplet regimen consisting of epirubicin, cisplatin, and 5-FU [4]. In contrast, the standard approach in Japan and Korea for patients with histologically confirmed stage II or III gastric cancer after D2 gastrectomy includes adjuvant chemotherapy with either S-1 [5] or capecitabine and oxaliplatin (XELOX) [6–9]. The JACCRO GC-07 trial further established the combination of S-1 and docetaxel as a standard adjuvant regimen for stage III gastric cancer in Japan, based on significantly improved recurrence-free survival (RFS) compared to S-1 monotherapy [10].

However, the regimen’s associated toxicities, including alopecia, neutropenia, and fatigue, along with the lower completion rates and economic considerations, have raised concerns regarding overtreatment, especially in older patients or those with comorbidities.

Among stage III patients, those classified as stage IIIA generally have a relatively better prognosis. It remains uncertain whether the intensified regimen with docetaxel is necessary for all patients in this subgroup. Therefore, identifying favorable prognostic factors in stage IIIA gastric cancer may help in tailoring adjuvant treatment intensity based on individual risk.

In this study, we retrospectively analyzed the clinical outcomes of stage IIIA gastric cancer patients who received S-1 monotherapy after curative gastrectomy, aiming to identify subgroups with favorable prognosis who may potentially achieve satisfactory outcomes without docetaxel. We emphasize that this investigation is exploratory in nature and not intended to challenge the current standard of care.

## Materials and methods

### Patients

**Fig. 1:**
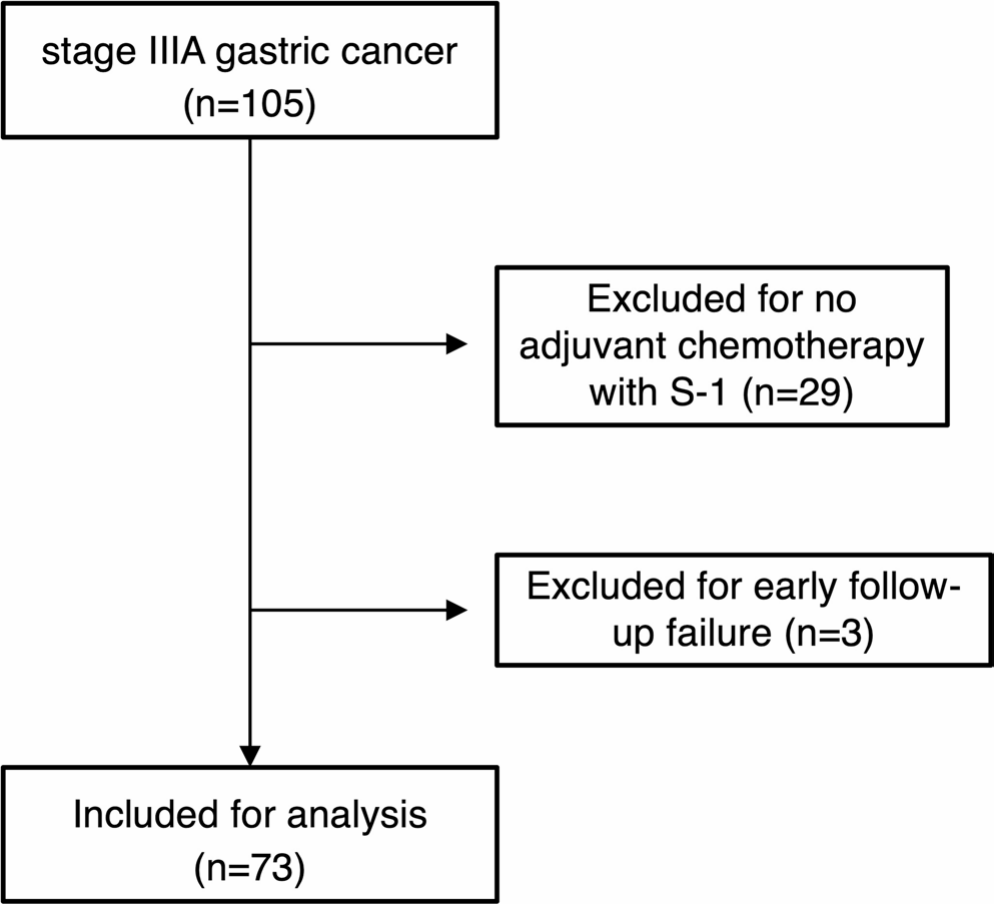
Consort diagram in this study

This retrospective cohort study analyzed a total of 105 patients who underwent curative gastrectomy at Hitachi General Hospital between June 2005 and March 2018. All patients were classified as having stage IIIA gastric cancer based on the 8th edition of the UICC TNM classification and the 3rd English edition of the Japanese Classification of Gastric Carcinoma (JCGC). Of these, 73 patients who received postoperative adjuvant chemotherapy with S-1 monotherapy were included in the study. Exclusions were made for 29 patients who did not undergo adjuvant chemotherapy and three patients with insufficient follow-up data (Fig. 1).

### Data collection and follow-up

Patient demographic and clinicopathological information was retrieved from the hospital’s electronic medical record system. Collected variables included age, sex, body mass index (BMI), operative duration, blood loss, T and N classification, tumor size, lymphatic and venous invasion, number of lymph nodes dissected, lymph node positivity rate (calculated as the ratio of metastatic nodes to dissected nodes), and histological type. Laboratory data obtained at least four weeks prior to surgery, including CEA and CA19-9 levels, were recorded alongside recurrence-free survival (RFS) data. Postoperative complications were assessed based on the Clavien–Dindo classification system [11] and cases with complications classified as grade 2 or higher were documented.

Patients were monitored through outpatient visits every 3–6 months following surgery. Cancer recurrence was evaluated via physical examination, thoracoabdominal computed tomography, and blood tests, including tumor marker measurements, conducted every six months.

### Statistical analysis

Receiver operating characteristic (ROC) curve analysis was utilized to identify optimal cut-off values for continuous variables, including BMI, operative duration, intraoperative blood loss, tumor size, number of dissected lymph nodes, and lymph node positivity rate. The determined thresholds were 22.2 kg/m² for BMI, 297 min for operative time, 212 ml for blood loss, 55.0 mm for tumor size, 47 lymph nodes for retrieval count, and 0.079 for lymph node positivity rate. Preoperative tumor marker thresholds were based on institutional upper normal limits, with CEA at 5.0 ng/ml and CA19-9 at 37 U/ml.

Univariate and multivariate analyses were conducted using the Cox proportional hazards model to identify prognostic factors associated with RFS. Kaplan–Meier survival analysis was performed to estimate RFS, and comparisons were made using the log-rank test. A significance level of *p* < 0.05 was applied. All statistical analyses were conducted using SPSS software version 27 (IBM, Chicago, IL, USA).

### Ethical conduct

This study was approved by the Research Ethics Committee of Hitachi General Hospital (acceptance number 2019 − 100). Because this was a retrospective, non-interventional study, the institutional review board had waived the need for written informed consent from the patient. All procedures carried out in the study involving human participants were in accordance with the ethical standards of the Institutional Research Board and the Declaration of Helsinki of 1964.

## Results

### Patient characteristics

The median follow-up period was 61 months (range: 3–106 months), during which 24 patients (32.9%) experienced tumor recurrence. Patient demographics and clinical characteristics are summarized in Table 1. The cohort included 55 male and 18 female patients, with a median age of 69 years (range: 51–85 years). Postoperative adjuvant chemotherapy was successfully completed within one year in 60 patients (82.2%). Dose reductions were required for 21 patients (28.7%): 18 patients (24.7%) underwent a single dose reduction, while 3 patients (4.1%) required two dose reductions. The 3-year and 5-year recurrence-free survival (RFS) rates were 71.7% and 64.3%, respectively, with a median RFS of 69 months. Among the surgical procedures, 70 patients (95.9%) underwent open gastrectomy, while 3 patients underwent laparoscopic surgery.

**Table 1:**
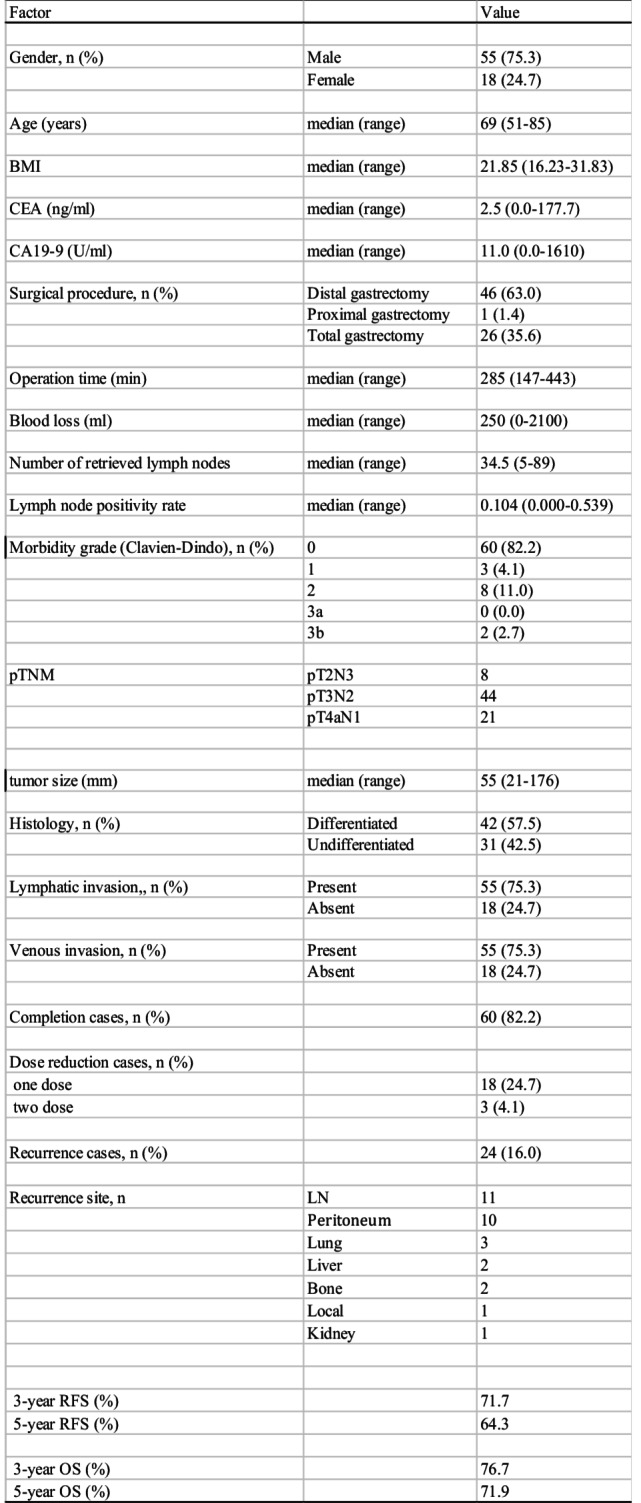
Clinicopathological characteristics of pStage IIIA gastric cancer patients

*BMI* body mass index, *CEA* carcinoembryonic antigen, *CA19-9* carbohydrate antigen 19 − 9, *LN* lymph node, *RFS* recurrence-free survival, *OS* over-all survival

### Favorable prognostic factors

Univariate analysis revealed that smaller tumor size (*P* = 0.026) and a lower lymph node positivity rate (*P* = 0.035) were significantly associated with longer RFS in patients with stage IIIA gastric cancer. Other factors, including T factor, N factor, lymphatic invasion, and venous invasion, showed no significant correlation with RFS (Table 2). Multivariate analysis confirmed that smaller tumor size (HR: 3.076; 95% CI: 1.380–6.855; *P* = 0.006) and a lower lymph node positivity rate (HR: 3.370; 95% CI: 1.369–8.296; *P* = 0.008) were independent prognostic factors for longer RFS (Table 3).

**Table 2:**
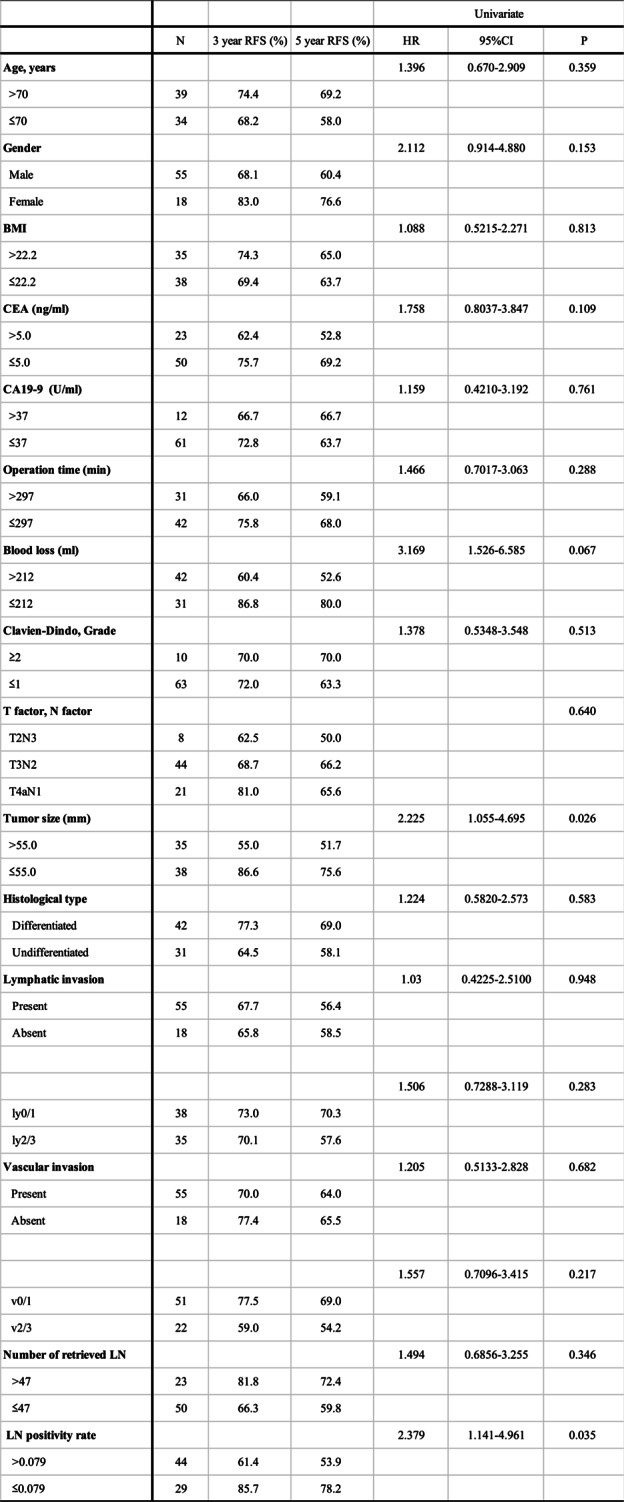
Univariate analysis of relationship between various clinical factors and RFS

*BMI* body mass index, *ng* nanograms, *ml* milliliters, *U* units, *min* minutes, *mm* millimeters, *LN* lymph node, *RFS* recurrence free survival

**Table 3:**
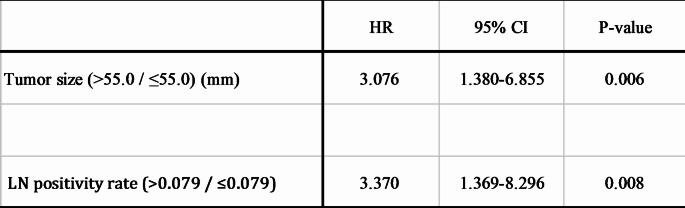
Multivariate analysis of prognostic factors for RFS

*LN* lymph node, *mm* millimeters

**Fig. 2:**
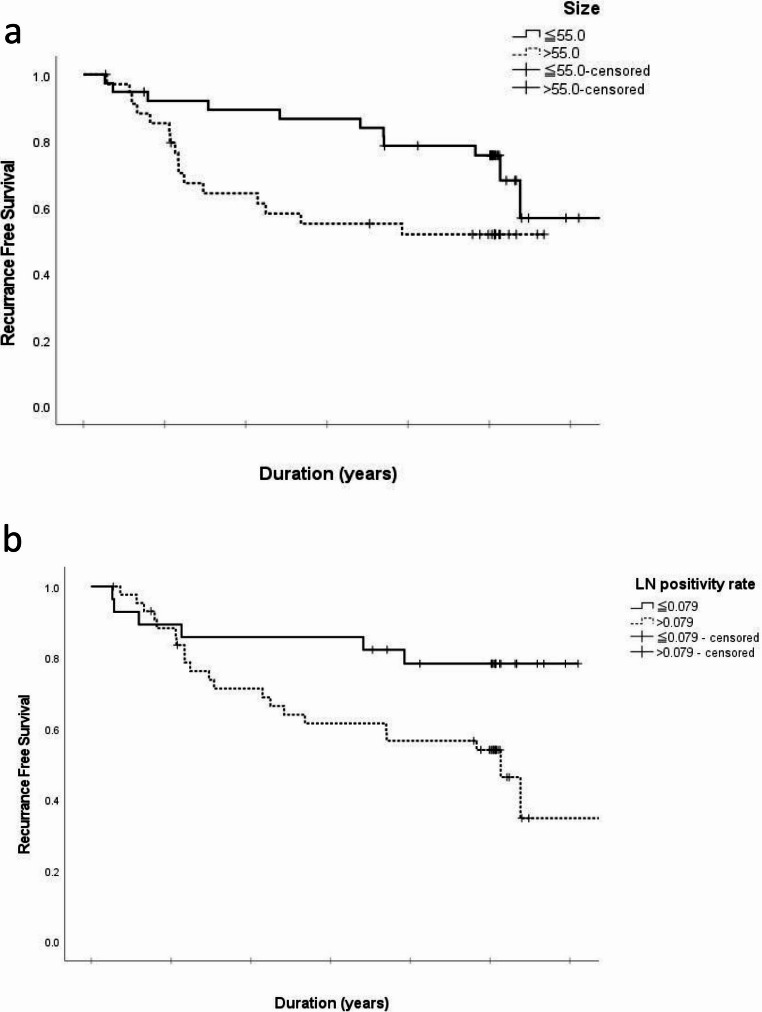
Kaplan–Meier survival graphs for recurrence free survival in relation to tumor size (2a), lymph node rate (2b): the figures show a lower recurrence-free survival with small tumor size and a low lymph node metastasis rate

Kaplan–Meier survival curves (Fig. 2a and b) stratified patients into groups based on a tumor size cutoff of 55 mm and a lymph node positivity rate cutoff of 0.079. Patients with smaller tumors had 3-year and 5-year RFS rates of 86.6% and 75.6%, compared to 55.0% and 51.7% in those with larger tumors (*P* = 0.026). Similarly, patients with a lower lymph node positivity rate demonstrated significantly better RFS, with 3-year and 5-year RFS rates of 85.7% and 78.2%, compared to 61.4% and 53.9% in those with higher rates (*P* = 0.035).

**Fig. 3:**
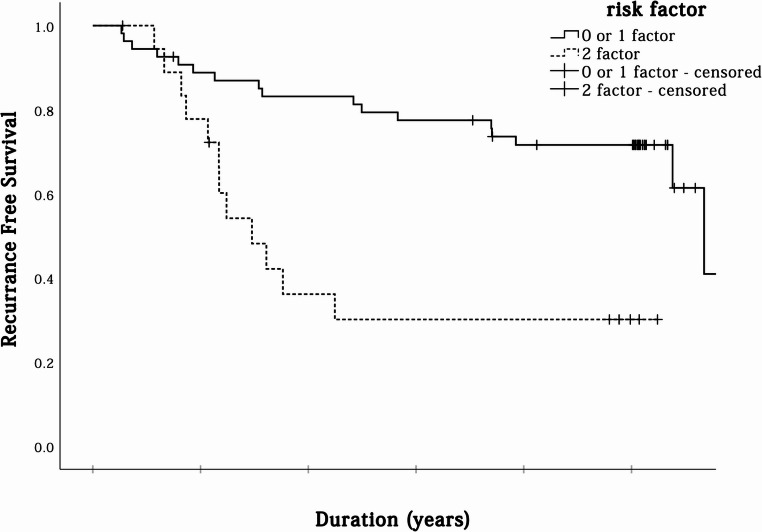
Kaplan–Meier survival graphs for recurrence-free survival in relation to tumor size and lymph node metastasis rate: the figures show a higher recurrence-free survival with large tumor size and high lymph node metastasis rates

Patients with both risk factors (large tumor size and high lymph node positivity rate) had notably poorer outcomes. Their 3-year and 5-year RFS rates were 30.1%, compared to 77.5% and 71.6%, respectively, in patients with zero or one risk factor (Fig. 3). The presence of two risk factors was associated with a significantly worse prognosis (*P* < 0.001; HR: 0.298).

## Discussion

In Japan, the JACCRO GC-07 trial established the combination of docetaxel and S-1 as the standard adjuvant chemotherapy for patients with stage III gastric cancer following curative resection [10]. While the feasibility of S-1 plus docetaxel therapy has been demonstrated [12]clinical trials report a docetaxel course completion rate of only 69%, with grade 3 or higher adverse events occurring in 58% of cases [10]. In actual clinical settings, particularly among elderly patients or those with comorbidities, the administration of docetaxel-based regimens may not always be feasible, raising concerns regarding overtreatment.

In this study, we retrospectively analyzed a cohort of patients with stage IIIA gastric cancer who underwent curative resection followed by S-1 monotherapy. We identified small tumor size and low lymph node positivity rate as independent favorable prognostic factors. Patients with these characteristics exhibited 3-year and 5-year recurrence-free survival (RFS) rates of 77.5% and 71.6%, respectively. These outcomes were comparable to the 5-year RFS reported for the S-1 plus docetaxel group in the JACCRO GC-07 study (75.10% for stage IIIA patients) [13]. 

However, we acknowledge that this retrospective, non-randomized analysis does not provide sufficient evidence to recommend S-1 monotherapy as a general alternative to the standard docetaxel-containing regimen. The observed favorable outcomes in our cohort may reflect selection bias or other confounding factors. Therefore, the present findings should not be interpreted as a basis for broadly endorsing S-1 monotherapy.

Instead, our results suggest that in selected patients—particularly those who are elderly, have significant comorbidities, or decline intensive treatment—S-1 monotherapy may be a clinically reasonable option. The identification of small tumor size and low lymph node positivity rate as favorable prognostic indicators may assist in treatment decision-making when docetaxel-based regimens are deemed unsuitable.

Previous studies have also highlighted tumor size as a critical prognostic factor. In a study involving 843 gastric cancer patients, Li et al. found that tumor diameter independently predicted poor outcomes, with a 1.317-fold higher risk of death for patients with tumors > 6 cm compared to those with tumors < 6 cm at 3 years [9]. Similarly, Kunisaki et al. identified a tumor diameter of 100 mm as a threshold for poor prognosis [14]. Larger tumors are frequently undifferentiated, prone to serosal invasion, and more likely to result in peritoneal recurrence, contributing to poorer outcomes.

Regarding lymph node metastasis, Erstad et al. analyzed 775 cases and found that examining ≥ 30 lymph nodes and a metastasis rate ≤ 30% were significantly associated with improved recurrence-free and overall survival [15]. According to Bilici et al., a retrospective analysis of 207 cases of pN3 gastric cancer reported that the lymph node metastasis rate, with a cutoff value of 0.75, was an independent prognostic factor for recurrence-free survival [16]. These findings support the role of the lymph node metastasis rate as a key prognostic factor in gastric cancer with comprehensive lymph node dissection. To obtain an accurate pN stage, the number of dissected lymph nodes is important; however, it may be influenced by specific surgical and pathological factors. To overcome these limitations, the lymph node metastasis rate has been reported in several studies as a novel prognostic factor [17]. Lymph node metastasis rate has been proven to be a good option for preventing the “stage migration” phenomenon and can accurately predict prognosis, particularly in patients with fewer than 15 dissected lymph nodes [18].

Past studies have pointed out the prognostic value of tumor size and lymph node metastasis rate. These simple pathological parameters are available postoperatively and may help clinicians stratify risk and tailor adjuvant treatment strategies. Nevertheless, prospective multicenter studies with larger sample sizes are needed to confirm whether S-1 monotherapy can provide adequate disease control in well-selected subgroups of stage IIIA gastric cancer patients.

The completion rate of the 8-course (12-month) regimen in this study was 82.2%, and 28.8% of patients required dose reduction in some form, indicating slightly better tolerability compared to JACCRO GC-07 (56% and 30%, respectively) [10]. In JCOG1104 (OPAS-1), which targeted stage II gastric cancer, it was reported that postoperative adjuvant chemotherapy with S-1 monotherapy for 12 months (8 courses) was more beneficial than a 4-course regimen [19]. The results of our study also highlight the importance of maintaining tolerability for the 12-month regimen.

This study has certain limitations. First, it is possible that adding docetaxel in patients with small tumors and low lymph node positivity rates could further improve outcomes. Second, as a retrospective, single-center study with a relatively small sample size, its findings may not be generalizable. As mentioned previously, future multicenter prospective studies involving larger cohorts are necessary to verify these results.

## Conclusions

In patients with resected stage IIIA gastric cancer, a small tumor size (≤ 50 mm) and a low lymph node positivity rate (≤ 0.079) were identified as independent favorable prognostic factors. These parameters may aid in risk stratification and treatment planning when standard regimens are unsuitable. Although S-1 monotherapy cannot be broadly recommended based on these results alone, it may be considered in selected low-risk patients. Further prospective studies are needed to confirm these findings.

## Data Availability

No datasets were generated or analysed during the current study.
